# Dermatological Brevities

**Published:** 1907-05-18

**Authors:** 


### DERMATOLOGICAL BREVITIES.

**The Temperature in Herpes.**

The analogy between herpes zoster and an acute exanthem is sometimes very striking. The temperature is nearly always raised, sometimes to as much as 101° on the first and second days of the appearance of the rash. With this pyrexia there is an accompanying feeling of malaise or even a slight rigor. After the third day, when no more vesicles have erupted, the temperature regains the normal.

**The Child's Skin.**

THE chief peculiarity about the treatment of skin disease in children is that the reaction to the remedies applied is more prompt than in adults. Moreover, since the risk of absorption is by no means inconsiderable, ointments and lotions containing powerful poisons, such as carbolic acid or mercury, should not be employed, unless well diluted, over large surfaces of the body. Certain cutaneous lesions are also very transient, so that one is left with their results, notably the scratchmark and the scab. The history of the mode of onset of an eruption, as given by an intelligent mother or nurse, is, therefore, of greater value than the statement of the patient himself, who might even be unaware of the existence of anything wrong with his skin.

